# Maternal aromatase inhibition via letrozole altered RFamide-related peptide-3 and gonadotropin-releasing hormone expression in pubertal female rats

**DOI:** 10.22038/IJBMS.2022.60962.13499

**Published:** 2022-01

**Authors:** Zahra Shaaban, Amin Tamadon, Mohammad Reza Jafarzadeh Shirazi, Mohammad Javad Zamiri, Amin Derakhshanfar

**Affiliations:** 1 Department of Animal Science, College of Agriculture, Shiraz University, Shiraz, Iran; 2 The Persian Gulf Marine Biotechnology Research Center, The Persian Gulf Biomedical Sciences Research Institute, Bushehr University of Medical Sciences, Bushehr, Iran; 3 Diagnostic Laboratory Sciences and Technology Research Center, School of Paramedical Sciences, Shiraz University of Medical Sciences, Shiraz, Iran; 4 Center of Comparative and Experimental Medicine, Shiraz University of Medical Sciences, Shiraz, Iran; # These authors contributed equally to this work

**Keywords:** Gonadotropin-releasing – hormone, Hypothalamus, Letrozole, Polycystic ovary syndrome, Prenatal, Rat, RFamide-related peptide-3

## Abstract

**Objective(s):**

Despite prevalence of polycystic ovary syndrome (PCOS) among childbearing women and development of many animal models for this syndrome, information on its etiology is still scarce. The intrauterine hyperandrogenic environment may underlie changes at the level of hypothalamus, pituitary, ovary organization in female offspring, and PCOS later in life. Letrozole has been shown to mimic reproductive and metabolic characteristics of PCOS in adult rodent models. Therefore, this research aimed to assess the condition in a prenatal letrozole-treated rat model.

**Materials and Methods:**

Twenty-eight female rats dams receiving letrozole at certain doses during late pregnancy were used in the trial. Pregnant Sprague-Dawley rats (n=21) received letrozole treatment on gestation days 16–18 at doses of 1.25, 1.0, 0.75, 0.5, and 0.25 mg/kg body weight (BW).

**Results:**

Prenatal letrozole treatment delayed parturition time and reduced the litter size in pregnant dams (*P*<0.0001). Late puberty onset, irregular ovarian cyclicity, increased anogenital distance (AGD), body weight gain, serum testosterone concentration, and reduced estradiol levels (*P*<0.0001) were observed in the female offspring of dams receiving 1.25 and 1 mg/kg BW letrozole. Furthermore, letrozole at 1.25 and 1 mg/kg BW showed increased RFRP and decreased GnRH mRNA expression (*P*<0.0001). Letrozole treatment at doses of 1 mg/kg BW and lower was not fetotoxic.

**Conclusion:**

It was concluded that 1 mg/kg BW letrozole may be suggested for prenatal PCOS induction.

## Introduction

Polycystic ovary syndrome (PCOS) is a gynecological disorder along with clinical or biochemical manifestation of hyperandrogenism and menstrual problems (1). Despite abundant information, there is a lack of a precise definition of its pathogenic mechanisms and animal models that accurately reflect the features of human PCOS. The underscored endocrine features of PCOS are hyperandrogenism and LH hypersecretion (2). According to the NIH, Rotterdam, and AE-PCOS conferences, hyperandrogenism is the most important diagnostic criteria of PCOS (3). Among androgenic animal models of PCOS, prenatal androgenized PCOS models displayed a set of reproductive and metabolic features of human PCOS (4). Androgen exposure during intrauterine life created a phenocopy of reproductive and metabolic features of PCOS in adult female rats (5). In pregnant women with PCOS, higher circulating levels of testosterone than in normal pregnant women were reported (6). Even though differentiation of hypothalamic centers regulating reproductive and ovarian activity (folliculogenesis and ovulation) occurs during fetal life (7), hormonal abnormalities during pregnancy can influence reproductive regulating centers in the hypothalamus. 

To create a hyperandrogenic environment during pregnancy, we used letrozole because it has the ability to mimic reproductive and metabolic characteristics as observed in PCOS, and also induces ovulation in PCOS women as a therapeutic drug. In addition, letrozole can affect the neuronal pathways in the brain that control gonadotropin secretion by disrupting the mechanism through which testosterone is converted to estrogen, and these effects can be transmitted epigenetically to the female fetus. Therefore, the use of these drugs in pregnant PCOS women may have adverse effects on their offspring in adulthood. Letrozole, a non-steroidal aromatase inhibitor, created hyperandrogenism and polycystic ovarian morphology in rats (8) and has been introduced as the second drug line for PCOS. Letrozole lacks peripheral anti-estrogenic effects and has a higher pregnancy rate than clomiphene citrate (9). Furthermore, letrozole is less costly than other ovulation-stimulating preparations, with a therapeutic efficacy similar to gonadotropin agonists (10). In a retrospective cohort study, the offspring of mothers treated with letrozole and clomiphene citrate had chromosomal abnormalities (11). Accordingly, we aimed to determine the optimal dose of letrozole for prenatal PCOS induction in female rats by assessing the reproductive, endocrine PCOS phenotypes and the hypothalamus-pituitary-gonad (HPG) axis alterations via evaluation of mRNA expression of *GnRH* and *RFRP* genes.

Up to now, a number of studies have highlighted that prenatal testosterone exposure disrupted HPG axis differentiation (12, 13). Recent evidence suggests that prenatal exposure to androgens leads to insensitivity of GnRH neurons to the negative feedback of steroids resulting in elevated LH levels in rodents, guinea pigs, and non-human primates (4). In addition, fetal androgen excess in rats affects the hypothalamic-pituitary axis and LH secretion (14). But there has been no model to induce PCOS using letrozole in the prenatal period so far. It is plausible that increased levels of maternal androgens by the mechanism of action of letrozole could cause changes in HPG axis differentiation including GnRH and RFRP-3 neuropeptides. Based on this previous finding, the question arose as to whether letrozole was able to produce a typical prenatal PCOS model; secondly, whether it would lead to changes in the hypothalamic level and major reproductive neuropeptides. Therefore, the present study was designed to determine the optimal dose of letrozole for prenatal PCOS induction in a rat model and to evaluate a number of endocrine, neuroendocrine, and reproductive features. 

## Materials and Methods

***Animals***

Adult (8-week-old) female Sprague-Dawley rats (n=30) weighing between 130 and 180 g were obtained from the Center of Comparative and Experimental Medicine, Shiraz University of Medical Sciences. The animals were kept in normal experimental condition at a 12-12 hr light-dark period, 23±3 °C, and 25±5% humidity, having free access to pelleted rat food and water.

After adaptation, vaginal smears were obtained from the rats by vaginal douching and the stages of the estrous cycle were determined based on the appearance of nucleated epithelial and cornified cells. The rats in proestrus or estrous phases (n=21) were joined with sexually experienced adult male rats (375 g mean weight) at a 2:1 female/male ratio overnight. The next morning, the rats were examined for the presence of the vaginal plug which was recorded as the sign of mating and the first day of pregnancy (gestation day: GD1).

***Prenatal letrozole induction of PCOS***

Pregnant rats were randomly divided into letrozole-treated, control, and sham groups. The optimal dose of letrozole was in a pilot trial designed to determine the safe doses of letrozole that showed no fetotoxic effects but could induce PCOS in the adult female offspring. Letrozole doses over 1.25 mg/kg resulted in parturition delay, fetal absorption, uterine infection, and pup mortality. 

Since our main goal was to induce changes in ovarian, endocrine, and neuroendocrine levels in female offspring to develop PCOS phenotypes, safe and non-lethal doses were selected. Twenty-one pregnant rats (n=3 for each group) were administered letrozole orally (L6545, Sigma-Aldrich, St. Louis, USA) dissolved in 1% carboxymethylcellulose (CMC, C5013, Sigma-Aldrich, St. Louis, USA) at concentrations of 0.25, 0.5, 0.75, 1.0, and 1.25 mg/kg BW during GDs 16, 17, and 18. In other words, letrozole doses were chosen to increase blood androgen levels higher than physiological levels that lead to PCOS development. Furthermore, GDs 16, 17, and 18 were chosen for letrozole administration owing to brain differentiation for inactivation of the LH surge center occurring in late rat pregnancy (15) and is practically an aromatase peak that occurs late in gestation and neonatal life (16). The sham group received vehicle only: 1% CMC dissolved in distilled water, and the control rats were untreated. Upon parturition, data including the parturition date, litter size, pup gender, and pup birth weight were recorded. The pups were kept with their mothers until weaning.

At weaning on postnatal day (PND) 21, the pups were sexed and weighed again. The anogenital distance (AGD) was measured using a caliper. The AGD index (AGDI) was calculated in order to normalize AGD for body weight at weaning. AGDI was calculated as AGD/BW × 100 (17). After weaning, female offspring were separated in each group (n = 4). The female pups (n = 4 per group from more than one dam), maintained under standard conditions, were weighed every two days from weaning until the end of the study. One-week post-weaning (on PND 28), the female offspring were checked for vaginal opening as the sign of puberty. Vaginal smears were evaluated from 28 PND for 4 weeks to determine the phases of the estrous cycle (18). 

***Tissue and blood sampling***

On postnatal day 49, four weeks after estrous cycle observation, chloroform (Merck, KGaA, index No, 602-006-00-4) was used for momentary anesthesia. The rats in each group were euthanized temporarily with a few drops of chloroform distributed on cotton in the desiccator. After that, blood was collected by cardiac puncture in tubes without anticoagulant, blood serum was prepared by centrifugation at 3000 rpm for 15 min. Blood serum was stored at -20 °C until evaluation of testosterone, estradiol, progesterone, follicle-stimulating hormone (FSH), and luteinizing hormone (LH).

Immediately after blood sampling and then cervical dislocation, the brain was removed from the skull, and was sampled for real-time PCR analyses of GnRH and RFRP-3 relative gene expression. The mammillary bodies were separated from the inferior and then optic chiasm from the posterior parts of the brain. Finally, with the third coronal cut, the diencephalon was removed and the pre-optic area (POA) and dorsomedial nucleus (DMN) were dissected in a piece of aluminum foil and immediately transferred into liquid nitrogen. Following decapitation, the ovaries were fixed in 10% buffer formalin. Blood and tissue sampling were performed regardless of the stage of the estrous cycle.

***Hormone analysis***

The serum concentrations of testosterone, estradiol, and progesterone were measured by competitive chemiluminescent enzyme immunoassay (IMMULITE 2000 System Analyzer, Cat. No. L2KTW2, L2KE22 and L2KPW2, respectively), and FSH and LH levels were determined by Rat FSH ELISA and Rat LH ELISA Kits via a sandwich enzyme immunoassay technique (Rainbow Biotechnologies Co., Cat. No. E0179Ra and E0182Ra, respectively).

***Histological evaluation***

The fixed ovaries were washed in phosphate-buffered saline (PBS), dehydrated in ascending concentrations of alcohol, and embedded in paraffin. Every ten serial sections (5 μm) were deparaffinized at 60 °C, transferred into ascending concentrations of ethanol and then xylene, and stained with hematoxylin and eosin (H&E). The number of total follicles including primary, secondary, tertiary, antral follicles and also atretic and cystic follicles, and corpora lutea were counted using a light microscope (CX21, Olympus, Japan) by one person blinded to the origin of the sections. For qualitative observations, three sections per animal from the beginning, middle, and end of the ovarian tissue were evaluated using a digital camera (Nikon, NI_U 2013, Japan).

***Expression levels of hypothalamic RFRP-3 and GnRH mRNA***

Total RNA from hypothalamic specimens was extracted according to the manufacturer’s extraction kit (Parstous RNA Isolation Kit, Parstous, Mashhad, Iran), and the pure extracted RNA was kept at -80 °C. The total purified RNA was measured by ultraviolet spectrophotometry (Nano-Drop, ND1000, USA). Removal of contamination was performed using the DNase I, RNase-free kit (Fermentas EN0521 DNaseI (RNase free)).

Based on the cDNA synthesis kit instructions (Smobio, Taiwan), the primers and dNTPs, as well as first-strand cDNA buffer, were mixed in a final volume of 20 µl, then incubated in 37 °C for 50 min and 85 °C for 5 min. Finally, for RNA removal, 1 µl RNase-H was added to each reaction medium and then incubated at 37 °C for 20 min. Prepared cDNA was preserved at -20 °C. To set up the annealing temperature, cDNA specimens were amplified by Thermocycler (StepOne™ Real-Time PCR, System Applied Biosystems, Carlsbad, CA, USA).

The primers for target genes *RFRP-3* and *GnRH* and the reference gene, β-*actin*, were designed by Primer3 software (Table 1). For each reaction of real-time PCR, a mixture of 10 µl SYBR Green (TaKaRa, Dalian, China), 7 µl distilled water, 1 µl forward primer, 1 µl reverse primer, and 1 µl cDNA was prepared in a final volume of 20 µl. Eventually, the amplification process was carried out by StepOnePlus Real-Time PCR Systems (Bioneer, South Korea) and the CT values were recorded by real-time PCR software. For evaluation of relative mRNA expression of *RFRP-3* and *GnRH*, the quantitative examination of CT values was carried out using the 2^-ΔΔCT^ formula.

***Statistical analysis***

Evaluation of data normality was conducted by the Kolmogorov-Smirnov test. For multiple comparisons among groups, one-way ANOVA and *post-hoc* Tukey were used. Binomial data were analyzed by the Chi-squared test. Multiple comparisons were carried out by the Kruskal-Wallis-H test for the data that were not normal. For weight gain, values from letrozole-treated, control, and sham groups were compared using repeated measures ANOVA. The differences among groups were considered statistically significant when *P*˂0.05. For data analysis, SPSS 22 for windows (IBM SPSS Statistics for Windows, version 22, IBM Inc., Chicago, Illinois, USA) was used. The data are reported as mean ± standard error of the mean (SEM). Charts were created by GraphPad Prism version 5.01 for Windows (GraphPad Inc. Inc., San Diego, CA, USA).

## Results

***Dam characteristics***

**Figure 1:**
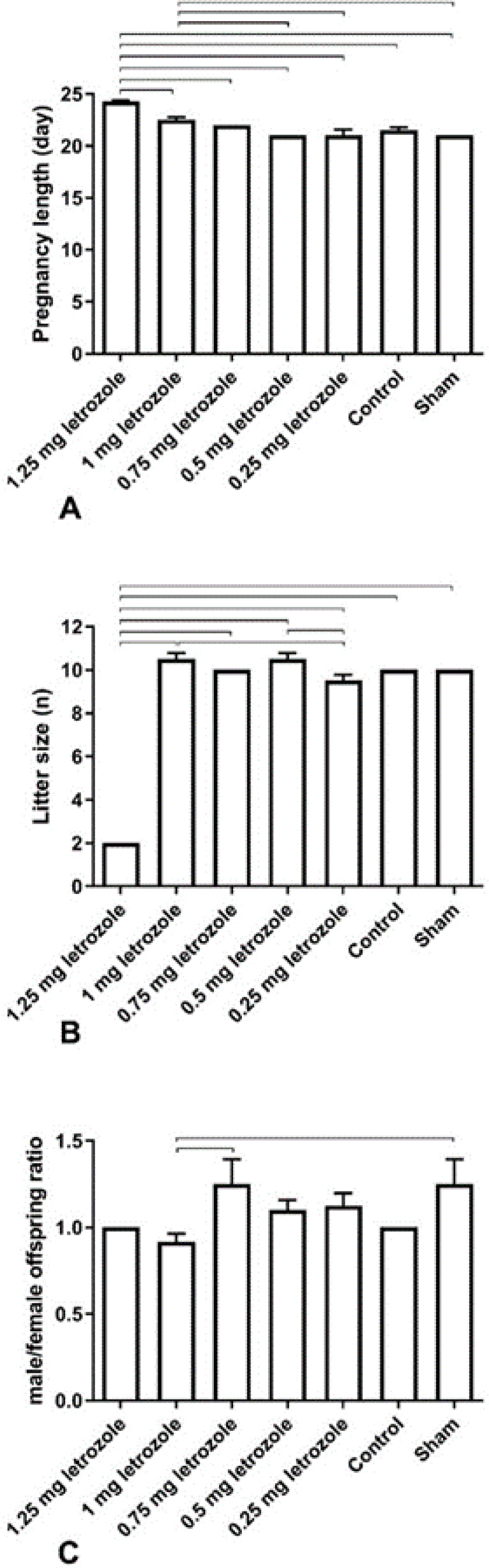
Prenatal letrozole treatment-induced effects on gestation length (A), litter size (B), and male/female offspring ratio (C) in adult rats (mean ± SEM). The lines show significant differences between groups at *P*<0.05

**Or**al administration of letrozole to rats on days 16, 17, and 18 of pregnancy impacted the gestation length (*P*<0.0001), number of neonates born (litter size) (*P*<0.0001), and male/female ratio (*P*=0.0369) in 1.25 and 1 mg/kg BW (Figures 1-A, B, C). A longer gestation length was observed in the 1.25 mg/kg BW group compared with all other groups. Furthermore, in the 1 mg/kg BW group, an increase in gestation length was observed compared with 0.5, 0.25 mg/kg BW, and sham groups (*P*=0.0093). In the pilot experiment evaluating the safe doses of letrozole in pregnant rats, doses higher than 1 mg/kg BW (1.5 to 3 mg/kg BW) on 16, 17, and 18 GDs delayed delivery noticeably (GDs: 26 days), and needed cesarean section (data not shown).

A significant decrease in litter size was observed at 1.25 mg/kg BW in comparison with other groups (*P*<0.0001). The pilot experiment also revealed that higher doses (1.5 to 3 mg/kg BW) of letrozole also reduced the litter size, and increased neonatal mortality and fetal absorption (data not shown). The offspring male/female ratio in the 1 mg/kg BW group was lowest among other groups, however, the difference was only statistically significant with sham and 0.75 mg/kg BW groups (*P*<0.05). 

***AGD and AGDI and puberty onset***

**Figure 2:**
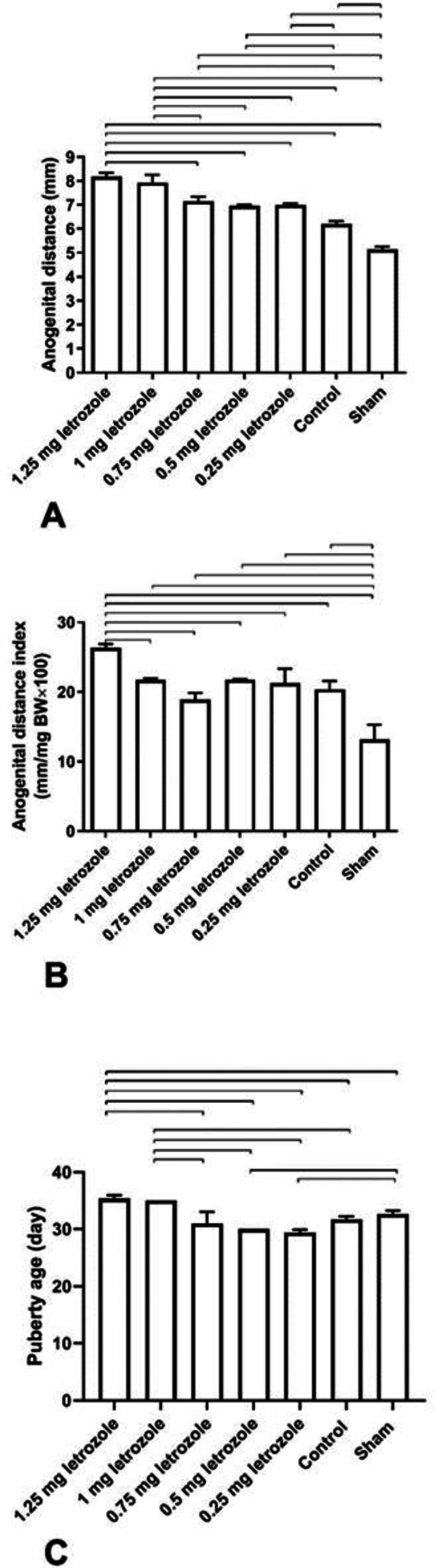
Prenatal letrozole treatment-induced effects on anogenital distance (AGD, A), AGD index (AGDI, B), and puberty age (C) in adult rats (mean ± SEM). The lines show statistically significant differences between groups at *P*<0.05

Prenatal letrozole treatment increased AGD in all experimental groups compared with control and sham groups dose-dependently (*P*<0.0001; Figure 2-A). Furthermore, AGDI was also higher in the 1.25 mg/kg BW group than in the other groups (*P*<0.0001; Figure 2-B), while, AGDI in the sham group was lower than in all other groups (*P*<0.0001; Figure 2-B). Delayed puberty was observed in 1.25 and 1 mg/kg BW groups (*P*<0.01; Figure 2-C).

***Bodyweight and ovarian weight***

**Figure 3:**
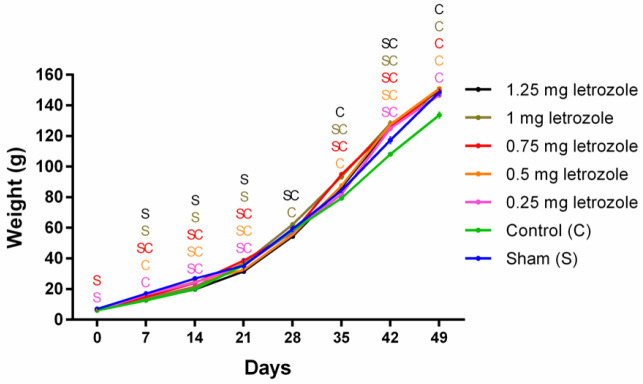
Prenatal letrozole treatment-induced effects on body weight gain in adult rats (mean + SEM). S (sham), C (control); The superscript letters show statistically significant differences between letrozole-treated groups with control and sham groups in each week (P<0.05)

Measurement of body weight from birth to 7 weeks showed that the time effect, letrozole effect, and their interaction were significant (*P*<0.0001). Letrozole treatment on 16, 17, and 18 GDs resulted in a significant increase in body weight gain at 5, 6, and 7 weeks compared with control and sham groups (*P*<0.01; Figure 3). In the sixth week, all letrozole-treated groups gained significantly more weight than the control group (*P*<0.0001; Figure 3). There was no effect of letrozole on the ovarian weight between groups on the sampling day (*P*=0.14).

***Estrous cycles***

**Figure 4.**
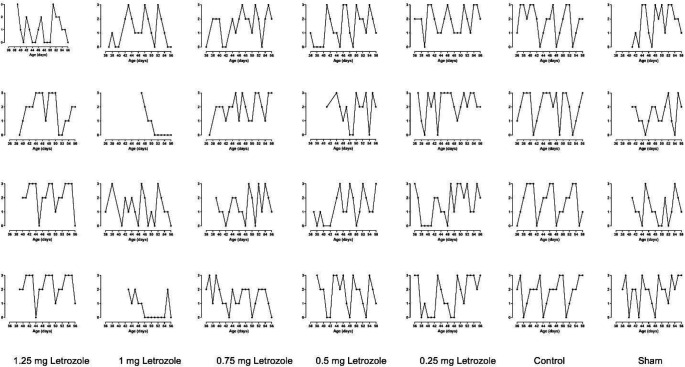
Prenatal letrozole treatment-induced effects on estrous cycle patterns during three week daily observations in adult rats. In the Y-axis, the numbers 3, 2, 1, 0 represent proestrus, estrus, metestrus, and diestrus

The evaluation of the estrous cycle during 20 days showed that the pattern of estrous cycles in letrozole-treated groups was irregular compared with other groups (*P*<0.0001; Figure 4). The absence of cycles and irregular cycles was observed at all doses of letrozole, and the greatest delay in the onset of cycles (first proestrus) after puberty was observed at 1.25 and 1 mg/kg BW doses which also recorded a delay in puberty as reported earlier. It is noteworthy that in control and sham groups, irregular estrous cycles were observed early in puberty and the cycles became more regular as puberty progressed (Figure 4). Moreover, estrous cycles in the sham group showed more irregularity than control, which was not statistically significant.

**Figure 5:**
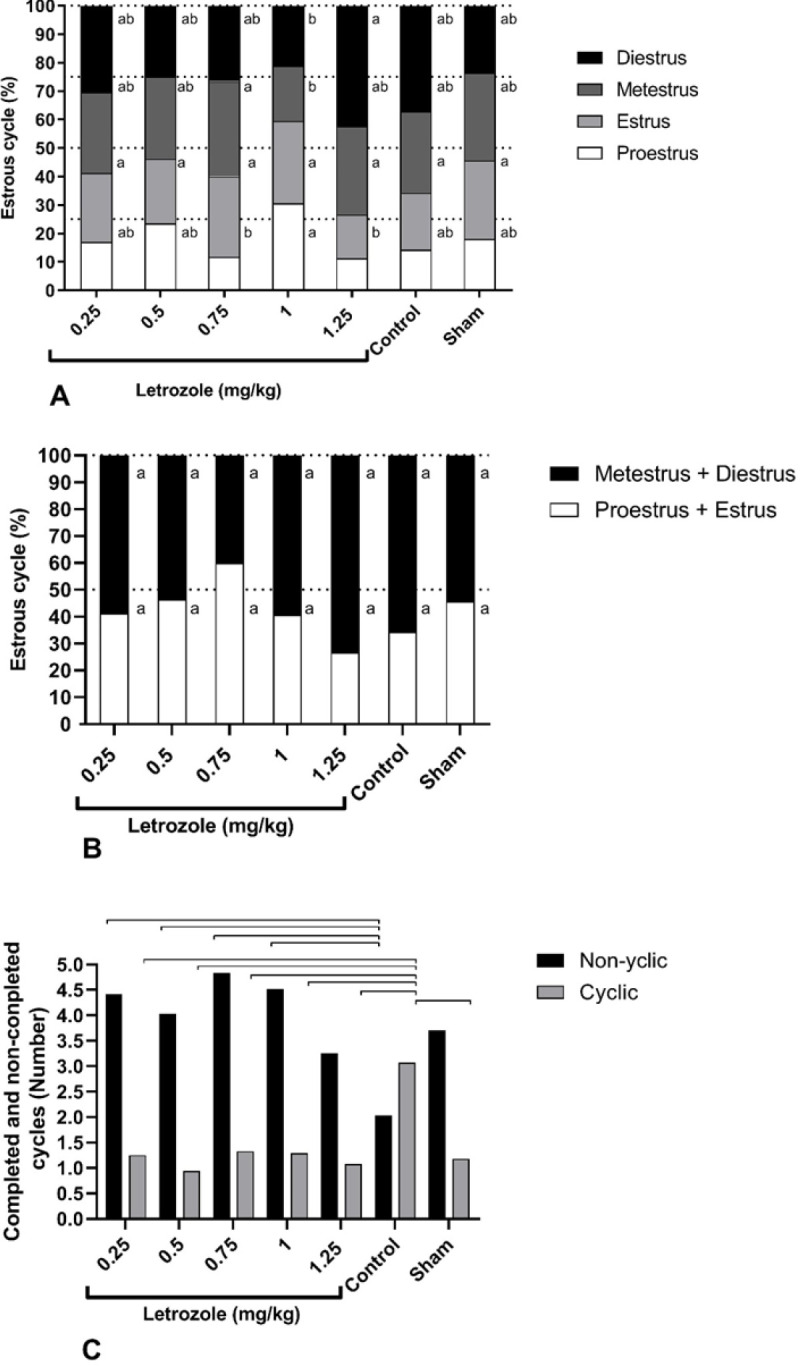
Prenatal letrozole treatment-induced effects on the percentage of each phase of the estrous cycle (A), total proestrus and estrous phases, and total metestrous and diestrous phases (B), and the number of completed and non-completed cycles (C) in letrozole-treated and control groups (mean + SEM). The mean with non-common letters has a statistically significant difference (*P*<0.05) in A and B. Also, the lines show statistically significant differences between groups at *P*<0.05 in C

The higher percentage of proestrus in the 1 mg/kg BW group was only significant compared with 0.75 and 1.25 mg doses (*P*<0.01). The percentage of metestrus at 1 mg dose was lower than at 0.75 mg dose, and the percentage of diestrus at 1.25 mg dose was higher than at 1 mg dose (*P*<0.01). There was no significant difference between the groups in the percentage of estrus (*P*=0.37; Figure 5-A). Furthermore, the sum of proestrus and estrus or follicular phase and the sum of metestrus and diestrus or luteal phases did not show a significant difference (*P*=0.9; Figure 5-B), while the sum of metestrus and diestrus in the 1.25 mg/kg BW group was higher than in all groups. The number of completed cycles (cyclic cycles) in the control group was higher than in other groups (*P*<0.05; Figure 5-C), on the other hand, the difference in non-completed cycles (non-cyclic cycles) between control and all other groups (*P*<0.05; Figure 5-C).

***Ovarian histology***

**Figure 6:**
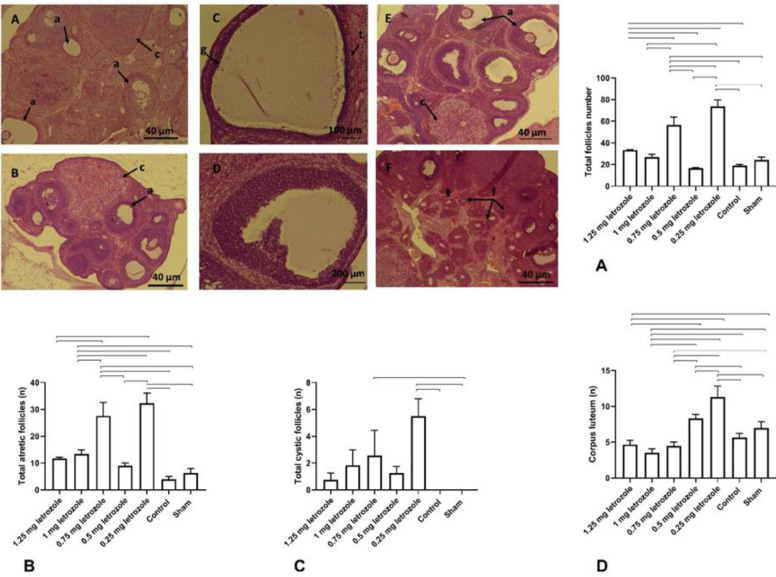
Prenatal letrozole treatment-induced effects on the number of total follicles (A), number of atretic follicles (B), number of cystic follicles (C), and number of corpus luteum (D) (mean + SEM), and H&E staining of rat ovaries in letrozole-treated and control groups. The total follicle number in the 0.25 mg/kg BW was more than o.75 and 0.5 mg/kg BW (A). Increased number of cystic and atretic follicles in 0.25 mg/kg BW compared with 0.5 mg/kg BW was shown in (B and C). Ovarian tissue in the control group (A) that has corpus luteum (c) and antral follicles (a) ovarian tissue in the 0.75 mg group (B), which has more antral follicles and corpus luteum (c), ovarian tissue in the 0.25 mg group (C), which has a large cystic follicle with a thin granulosa layer (g) and theca layer (t), large cyst in the 0.75 mg group (D), ovarian tissue in the 0.25 mg group (E) has a large number of antral follicles (a) as well as corpus luteum (c), ovarian tissue in 1 mg group (F), which has a large number of growing follicles (f) and antral follicles. The lines show statistically significant differences between groups at *P*<0.05

A significant increase in the total number of primary, secondary, antral, and atretic follicles, and corpora lutea were observed in the 0.25 mg/kg BW group compared with all other groups (*P*<0.0001; Figures 6- A, B-D). Although the 1.25 mg/kg BW treatment resulted in elevation of total follicle number compared with control (*P*=0.013; Figure 6-A), in atretic, cystic, and corpora lutea no significant difference was observed. Instead, in 1 mg/kg BW significant increase in atretic follicles and a decrease in corpora lutea were shown in comparison with control and sham groups (*P*<0.01; Figures 6-B, D). However, the number of cystic follicles in 0.25 and 0.75 mg/kg BW groups was higher than in sham and control rats (*P*<0.01; Figure 6-C).

***Serum concentration of steroids and gonadotropins***

**Figure 7:**
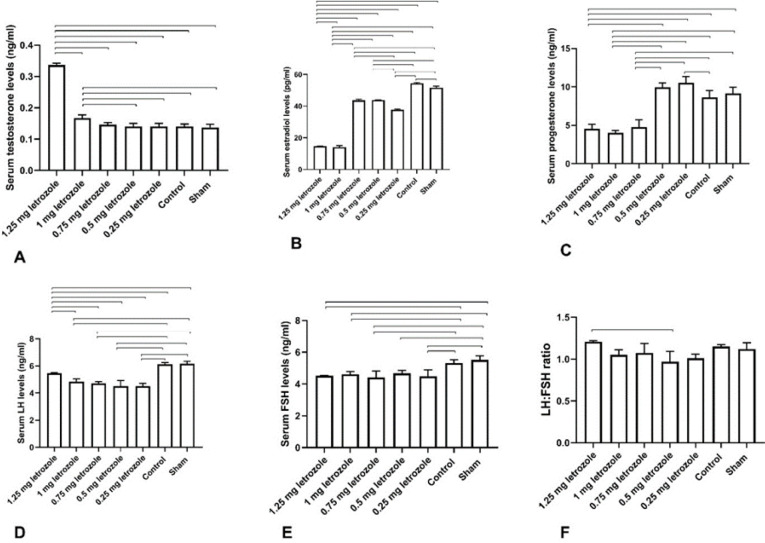
Prenatal letrozole treatment-induced effects on serum concentration of testosterone (A), estradiol (B), progesterone (C), LH (D), FSH (E), and LH: FSH ratio (F) (mean + SEM) in adult rats. The lines show statistically significant differences between groups at *P*<0.05

Letrozole treatment at doses of 1.25 and 1 mg/kg BW elevated serum testosterone concentration remarkably (*P*<0.0001; Figure 7- A). On the other hand, serum estradiol levels in 1.25 mg and 1 mg groups were lower than in other groups (*P*<0.0001; Figure 7- B). Serum progesterone levels in 1.25, 1, and 0.75 mg/kg BW did not show significant differences, however, these groups had lower progesterone levels than the other groups (*P*<0.0001; Figure 7- C). 

Letrozole treatment of female rats resulted in lower serum concentration of LH (*P*=0.001; Figure 7- D) and FSH (*P*=0.005; Figure 7- E) compared with control and sham groups. Furthermore, there was no significant difference in LH: FSH ratio, except between 1.25 and 0.5 mg/kg BW groups (*P*=0.014; Figure 7- F).

***Gene expression of hypothalamic polypeptides RFRP-3 and GnRH***

**Figure 8:**
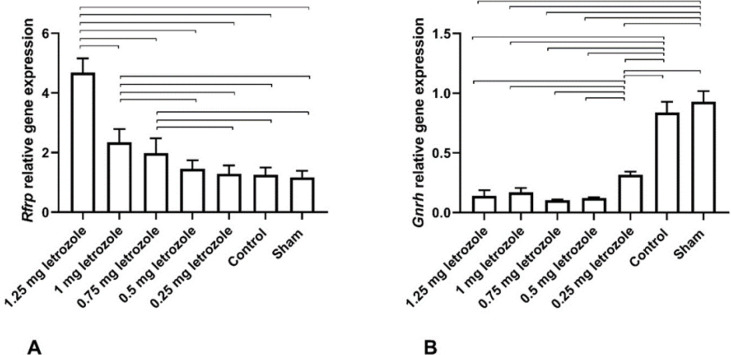
Prenatal letrozole treatment-induced effects on relative gene expression of hypothalamic neuropeptides, Rfrp (A) and GnRH (B), (mean + SEM) in adult rats. The lines show statistically significant differences between groups at *P*<0.05

The relative expression of the hypothalamic RFRP-3 gene increased with increasing dose of letrozole, being the highest at 1.25 mg dose; however, hypothalamic GnRH gene expression decreased due to letrozole treatment (*P*<0.0001; Figure 8). Increased RFRP expression in 1.25, 1, and 0.75 mg/kg BW was observed compared with other groups (*P*<0.05). On the other hand, decreased GnRH expression was shown in all letrozole-treated groups (*P*<0.0001). 

## Discussion

Decreased *GnRH* gene expression as a result of prenatal letrozole treatment in female rats was proven in this study for the first time. Letrozole-induced mouse model of PCOS showed increased expression of gonadotropin-releasing hormone receptor (*GnRhr) *in the pituitary, which was not reversible by flutamide treatment (16). Moreover, increased hypothalamic *GnRH* and pituitary *Gnrhr* transcripts were observed in an adult rat model induced by 21 days 0.5 mg letrozole administration (19). In another adult letrozole PCOS model, no changes in *GnRH *mRNA expression, but increased pituitary *Gnrhr* mRNA expression was observed (20). Moreover, an elevated number of AR and GnRH immunoreactive cells and *AR* mRNA expression were shown due to DHT-induced PCOS in adult rats (21). The lack of prenatal androgenization PCOS studies evaluating *GnRH* expression, and inconsistencies in studies of PCOS induction with letrozole in adulthood complicate the interpretation of results. However, the mechanism that indicates an increase in *Gnrhr* expression appears to be more influential in the etiology of PCOS (20). 

On the other hand, puberty onset is controlled by high-frequency GnRH neurons that affect FSH and LH release to trigger gonads for puberty initiation (22); therefore, the elevated *GnRH* expression in our study in the untreated group may be due to the normal neuroendocrine changes at puberty onset. At the same time, decreased *GnRH* expression in letrozole treated groups led to late puberty. However, it would be better to investigate the direct effect of androgens using flutamide during pregnancy on these neuroendocrine changes.

In addition, the impact of upstream mechanisms on GnRH release should not be overlooked. Letrozole-treated adult female mice revealed increased neuronal activation of *Kiss1* (23). A DHT-induced PCOS rat model resulted in decreased *kiss1* gene expression, but the serum levels of testosterone, estradiol, LH, FSH were unaltered (24). Interestingly, in a letrozole-induced PCOS model in adult rats, increased positive-cell number of kisspeptin in the arcuate and decreased the number of positive kisspeptin in anteroventral periventricular (AVPV) nucleus were reported (25). Kisspeptin neurons in the arcuate nucleus are involved in the negative feedback of estradiol on the GnRH/LH system. On the other hand, kisspeptin neuropeptides of the AVPV mediate preovulatory LH surge (26). Therefore, increased *kiss1* gene expression in the arcuate nucleus can interfere with the PCOS pathology (25). In general, the effect of prenatal and adult administration of androgens on inhibitory and excitatory regulators of GnRH neurons is likely more important than the direct impact of androgens on these neurons. In various models, alterations in *Gn*RH expression or basal or pulsatile levels of GnRH neuropeptide are different. This might be due to the fact that different neural pathways influence the GnRH neurons (23). Although, there are limitations due to the lack of information on GnRH pulse frequency and amplitude, so the baseline value of serum LH levels cannot be attributed to the pulsatile GnRH secretion and also *GnRH *gene expression. Given the fact GnRH secretion is affected by a set of stimulatory and inhibitory factors, including KNDy neuropeptides and gamma-aminobutyric acid (GABA)ergic neurons, further studies are needed to evaluate the changes in upstream GnRH regulators, especially in prenatal PCOS models.

Compared with other groups, increased expression of the *RFRP* gene in the hypothalamic DMN was observed at 1.25 and 1 mg/kg BW letrozole treatment. Prepubertal letrozole implants releasing 50 µg/day for 16 days before puberty did not impact *RFRP* expression in DMN; however, increased LH levels were found in adult female rats suggesting the role of other endogenous regulators of GnRH such as KNDy neuropeptides or GABAergic inputs rather than RFRP-3 neurons (23). Moreover, in our previous study, the constant light-induced PCOS rat model decreased *RFRP* expression, along with unaltered FSH and LH serum levels in adult female rats (27). Furthermore, in neonatal testosterone-treated female rats, decreased *RFRP* mRNA expression was reported, without any effect on LH serum level (28). These studies revealed that serum LH concentration did not reflect the effect of *GnRH* and *RFRP* expression changes directly. In other words, the changes in the expression of these neuropeptides are probably not the main neuroendocrine mechanism for LH increase in PCOS women. On the other hand, it was shown that intracerebroventricular injection of RFRP-3 decreased *GnRH* mRNA expression in female rats (22), demonstrating the inhibitory effect of RFRP-3 on GnRH neurons. Therefore, alteration in *RFRP* expression in control groups is normal for the ages after puberty onset, but in the treated group, especially at 1.25 and 1 mg/kg BW, enhanced *RFRP* expression may be a result of excessive androgen production during intrauterine life.

Taken together, given that the intrauterine hyperandrogenic environment is a critical etiological agent in neurodevelopmental diseases and also hyperandrogenism status in PCOS women. Although our study addressing the main purpose of assessing neuroendocrine changes specific to PCOS was not straightforward, but this study is a background for future assessments.

This work highlighted delayed puberty as a result of 1.25 and 1 mg/kg BW prenatal letrozole treatment. Commonly, vaginal opening in rats occurs at 28–49 postnatal days (PNDs), but the age of puberty onset and beginning of sexual maturation is different according to the species and growth rate (29). Delayed puberty in our study, maybe due to increased *RFRP *expression in 1.25 and 1 mg/kg BW groups. Similarly, it was shown that intracerebroventricular injection of RFRP-3 between days 28 and 36, at the time of puberty onset, delayed vaginal opening in rats (22). This reduces its inhibitory effect on GnRH neurons (30). Similarly, GPR147 (the RFRP-3 receptor) knockout on GnRH neurons caused delayed puberty in mice (30). The increased expression of *RFRP* may be due to elevated androgen levels during the intrauterine life but this hypothesis should be investigated further by evaluating AR on RFRP-3 neurons or by blocking the direct effects of androgen by flutamide. 

The findings of our research showed markedly elevated testosterone levels and reduced estradiol levels in 1.25 and 1 mg/kg BW groups. The increased endogenous androgen production by inhibiting aromatase function is unavoidable and has been reported in different models of PCOS induction with letrozole (19, 31, 32). In daughters of women with PCOS, increased testosterone levels were reported during puberty but in general, there is little information available (33). reduced estradiol levels were reported in the letrozole treated PCOS adult rat model (32, 34). Given that the estradiol levels ​​vary during the estrous cycle, and estradiol is at the lowest level in the estrous phase, these differences are probably due to measurements taken at different times during the estrous cycle. On the other hand, variations in estradiol levels are the direct effects of letrozole due to reducing the conversion of testosterone to estradiol. 

Serum FSH and LH levels were also higher in control groups showing that gonadotropins levels were not affected by prenatal androgenization via letrozole. Letrozole treatment in adult female rats for 14 days resulted in reduced estradiol levels (35). Increased LH levels, as the main feature of PCOS women, have also been observed in adult rodent letrozole models (20, 36, 37). In the present study, serum LH levels at 1.25 mg/kg BW were increased compared with other groups. In prenatal androgenized female rats, FSH levels were not affected by androgen action (38, 39). There have been inconsistencies in the baseline and pulse concentrations of LH and FSH in various PCOS prenatal and clinical studies of PCOS women (40-42). These inconsistencies may be due to the different estrous phases of female rats during sampling, animal models and species, time of induction, and other experimental conditions. 

In the current study, all letrozole treated groups exhibited acyclicity. Moreover, the delay in initiation of cycles (first proestrus) and reduced number of females that were able to complete one or more cycles were observed in all treated groups. Irregular cyclicity was observed as a result of prenatal letrozole administration. Irregular cycles and ovulation dysfunction are the usual effects of exposure to androgen excess during intrauterine life (43, 44). Increased total numbers of follicles and atretic follicles were observed in 1.25 and 1 mg/kg BW, respectively. It seems that prenatal administration of letrozole has not been able to develop the morphological characteristics of PCOS as well as the adult PCOS model of letrozole (8). Increased numbers of antral and preantral follicles were observed in prenatal rats exposed to androgen on 16-19 GDs (39), indicating that the timing of androgen administration is important. These findings suggest that, first, the prenatal hyperandrogenic letrozole model is unable to produce morphological features of PCOS; and, second, that this trait may not be affected by the intrauterine environment.

The letrozole androgenized female rats showed longer AGD at puberty than untreated groups. Furthermore, we observed increased AGD in all treated groups. AGD is longer in the male rats than in the female ones, so ADG in females reflects the degree of uterine hyperandrogenism that she experienced during the intrauterine life, and the higher doses of androgen exposure during fetal life led to longer AGD in female offspring. In addition, the long AGD in prenatal T and DHT (on 16-19 GDs) female rats was shown (38). Increased AGD reflects the androgenic effect of letrozole during the time of external genitalia differentiation (19–20 GDs). These findings suggest that androgen action during embryonic life, especially at the time of external genitalia differentiation, affects AGD, which is a good indicator of a direct effect of androgens. 

Prenatal letrozole administration at 1.25, 1.0, and 0.75 mg/kg BW significantly increased the bodyweight gain compared with other groups. These bodyweight gain increments were greater than those of control groups at 6 to 8 postnatal weeks, suggesting the metabolic effects of prenatal letrozole appeared at adulthood. Increased bodyweight gain as a metabolic feature of PCOS has been confirmed in various adult letrozole-induced PCOS models in rats (8, 34, 45, 46). The prepubertal letrozole model caused increased body weight gain in mice which did not improve with letrozole removal, contrary to the reproductive traits induced in this model that was completely recovered (47). However, in another study, weight gain caused by the adult letrozole PCOS model was reversible by flutamide in mice (48). These findings suggested that metabolic alterations such as bodyweight gain by prenatal or prepubertal origins were more permanent. 

Delayed delivery observed in treatments with 1.25 and 1 mg/kg BW letrozole may be due to the reduced level of estrogen induced by letrozole treatment. High levels of estrogen are necessary for initiation of parturition (35). Because estrogen is greatly increased in the last third of rat pregnancy (49) and rodent ovaries are the prominent site of estrogen biosynthesis throughout pregnancy (35), inhibition of estrogen synthesis in the ovary in late pregnancy causes longer gestation length. Letrozole administration at 0.002 or 0.02 mg/kg BW per day during 15- 21 GDs delayed parturition and reduced litter size in rats (50). Reduced litter size was observed in the 1.25 mg/kg group, suggesting that letrozole at high doses has severe fetotoxic effects. Letrozole administration during organogenesis (6–16 GDs) in rats at 0.01, 0.02, and 0.04 mg per kg doses resulted in a post-implantation loss that included early and late resorption and decreased the number of viable fetuses (51). These findings suggested that embryo and fetotoxic effects of letrozole were dose-dependent, depending on the time of administration (35). It seems these changes are caused by letrozole action in reducing estrogen levels because simultaneous estrogen treatment at the maximum dose of letrozole (0.04 mg/kg BW) restrained the fetotoxic effects of letrozole (52). However, increased fetal mortality may be due to the direct effect of androgen increment as a result of letrozole, as PCOS women have been reported to have higher perinatal fetal mortality rates than women with normal androgen levels (53). Furthermore, the shorter exposure time and higher letrozole dose was the main difference between our study and these studies; so it seems that the disrupting effects of letrozole are related to the specific (organogenesis or fetal stage) time during pregnancy (35) and the administrated dose. 

**Table 1:** Real-time PCR primers for target and reference genes and annealing temperatures

Gene	Sequence		Product size (bp)	Annealing temperature (°C)
GnRH	Forward	5'-AATACTGAACACTTGGTTGA-3'	20	57
	Reverse	5'-AGATCCCTAAGAGGTGAA-3'	18	
RFRP-3	Forward	5'-AAGACACTGGCTGGTTTG-3'	18	56
	Reverse	5'-TTGAAGGACTGGCTGGAG-3'	18	
β-actin	Forward	5'-CACAGCTGAGAGGGAAAT-3'	18	56
	Reverse	5'-TCAGCAATGCCTGGGTAC-3'	18	

## Conclusion

In summary, the results of our study suggested that prenatal letrozole treatment at doses lower than 1 mg/kg BW is safe and has no detrimental embryotoxic or lethal effects on fetuses and mothers in Sprague-Dawley rats. A comprehensive dose-response study was carried out to evaluate the alterations of major genes controlling reproductive phenomena,* GnRH* and *RFRP*, steroid hormones and gonadotropins, and ovarian function in response to prenatal letrozole administration. We recorded irregularities in estrous cycles, follicular development changes, increased testosterone levels, decreased estradiol levels, decreased *GnRH,* and increased *RFRP* expression at 1.25 and 1 mg/kg BW letrozole. Due to the greater effect of 1.25 mg/kg BW treatment on fetal mortality, we finally concluded that prenatal letrozole treatment at 1 mg/kg BW on 16–18 GDs in rats, is probably optimal for PCOS induction by inhibiting aromatase and indirectly increasing endogenous androgen levels. In terms of future work, it may be best to first confirm the prenatal PCOS letrozole model at a dose of 1 mg/kg BW. Furthermore, it would be interesting to examine the upstream and downstream pathways that control the GnRH and RFRP-3 neurons. 

## Authors’ Contributions

ZS Carried out the experimental works, performed the analytical methods, and wrote the draft of the manuscript. AT Conceived the original idea, supervised the project, performed the analytical methods, designed the figures, and wrote the final version of the manuscript. MRJS Provided the grant of the project, supervised the project, and contributed to the final correction of the manuscript. MJZ Consulted the thesis project and contributed to the final correction of the manuscript. AD Contributed to the final correction of the manuscript. All authors read and approved the final manuscript.

## Funding

This work was supported financially by Shiraz University (grant no. 97gcu3m148075 for PhD students).

## Ethics Approval

All procedures were approved by the Ethics and Research Committee of Shiraz University (Approval ID: IR.SUMS.REC.1397.434; Approval date: 2018-08-01), and carried out in accordance with the health instructions for the care and use of animals.

## Consent to Participate

Not applicable.

## Availability of Data and Material

The authors confirm that the data supporting the findings of this study are available within the article and raw data supporting the findings of this study are available from the corresponding author upon request.

## Conflicts of Interest

The authors declare that the research was conducted in the absence of any commercial or financial relationships that could be construed as a potential conflict of interest.
